# Live imaging of Aiptasia larvae, a model system for coral and anemone bleaching, using a simple microfluidic device

**DOI:** 10.1038/s41598-019-45167-2

**Published:** 2019-06-25

**Authors:** Will Van Treuren, Kara K. Brower, Louai Labanieh, Daniel Hunt, Sarah Lensch, Bianca Cruz, Heather N. Cartwright, Cawa Tran, Polly M. Fordyce

**Affiliations:** 1 Department of Microbiology and Immunology, Stanford University, Stanford, CA 94305 USA; 2 Department of Bioengineering, Stanford University, Stanford, CA 94305 USA; 3 Department of Chemical Engineering, Stanford University, Stanford, CA 94305 USA; 4 Department of Genetics, Stanford University, Stanford, CA 94305 USA; 5 Department of Physics, California State Polytechnic University, Pomona, CA 91768 USA; 6 Department of Plant Biology, Carnegie Institution for Science, Stanford, CA 94305 USA; 7 Chem-H Institute, Stanford University, Stanford, CA 94305 USA; 8 Department of Biological Sciences, California State University, Chico, CA 95929 USA; 9 Chan Zuckerburg BioHub, San Francisco, CA 94158 USA

**Keywords:** Lab-on-a-chip, Coral reefs, Biomedical engineering

## Abstract

Coral reefs, and their associated diverse ecosystems, are of enormous ecological importance. In recent years, coral health has been severely impacted by environmental stressors brought on by human activity and climate change, threatening the extinction of several major reef ecosystems. Reef damage is mediated by a process called ‘coral bleaching’ where corals, sea anemones, and other cnidarians lose their photosynthetic algal symbionts (family Symbiodiniaceae) upon stress induction, resulting in drastically decreased host energy harvest and, ultimately, coral death. The mechanism by which this critical cnidarian-algal symbiosis is lost remains poorly understood. The larvae of the sea anemone, *Exaiptasia pallida* (commonly referred to as ‘Aiptasia’) are an attractive model organism to study this process, but they are large (∼100 mm in length, ∼75 mm in diameter), deformable, and highly motile, complicating long-term imaging and limiting study of this critical endosymbiotic relationship in live organisms. Here, we report ‘Traptasia’, a simple microfluidic device with multiple traps designed to isolate and image individual, live larvae of Aiptasia and their algal symbionts over extended time courses. Using a trap design parameterized via fluid flow simulations and polymer bead loading tests, we trapped Aiptasia larvae containing algal symbionts and demonstrated stable imaging for >10 hours. We visualized algae within Aiptasia larvae and observed algal expulsion under an environmental stressor. To our knowledge, this device is the first to enable time-lapsed, high-throughput live imaging of cnidarian larvae and their algal symbionts and, in further implementation, could provide important insights into the cellular mechanisms of cnidarian bleaching under different environmental stressors. The ‘Traptasia’ device is simple to use, requires minimal external equipment and no specialized training to operate, and can easily be adapted using the trap optimization data presented here to study a variety of large, motile organisms.

## Introduction

Coral reefs are remarkably productive ecosystems, supporting approximately 9% of the ocean fish biomass and 25% of oceanic species diversity^1^. Reef-building corals depend on an endosymbiotic relationship with dinoflagellate algae (family Symbiodiniaceae) for survival and productive growth^2,3^, as most corals and other symbiotic cnidarians derive their primary metabolic energy through algal photosynthesis^4^. Under environmental and anthropogenic stressors such as rising ocean acidity, pollution, and increasing temperature, the coral-algal symbiotic relationship can break down, resulting in a process known as‘coral bleaching’^1,5^. In this process, photosynthetic algal symbionts are expelled from the coral gastrodermal tissue where they normally reside, resulting in coral discoloration and, if prolonged, host death due to insufficient energy metabolism^5^. On a macro-scale, coral bleaching has been implicated as a major cause of rapid worldwide reef deterioration and ecosystem disruption^1^. To address this threat, coral-algal symbiosis, in general, and the mechanism of coral bleaching, in particular, must be better understood.

While candidate stressors that contribute to coral bleaching are known, the molecular and cellular mechanisms of algal expulsion remain poorly characterized, in part due to the difficulties of working with corals in the laboratory^3,6^. In this work, we use a small sea anemone, *Exaiptasia pallida* (commonly referred to as Aiptasia), as a model system for coral and anemone symbiosis^6–9^. Aiptasia is an attractive organism for mechanistic biology studies of coral because both larval and adult animals are compatible with laboratory culture and algal infection^6^. Aiptasia larvae, in particular, enable study of the earliest stages of coral symbiosis because the larval stage is the earliest developmental stage in which cnidarians can take up algal symbionts^2,6,10^.

To date, live imaging of Aiptasia larvae has proven experimentally intractable at any throughput. Powerful cilia make Aiptasia and other cnidarian larvae highly motile (Movie S1). Because no chemical, genetic, or pharmaceutical tools are currently available to arrest ciliary movement in living cnidarian and marine animals, live imaging of fully motile animals is required to study dynamic organism physiology and behavior. However, researchers have not been able to observe symbiosis induction, maintenance, or bleaching within the same live larva over time, even for a single larva, limiting basic understanding of the critical cnidarian-algal endosymbiotic relationship^11^.

Painstaking efforts to culture larvae in the laboratory^5,12^, characterize the genome^8^ and transcriptome^7,9^, and perform phenotypic imaging^13^ have established Aiptasia as a new model organism^3,6,8,11^. Study of algal symbiosis in Aiptasia (and other cnidarians) previously has relied on adult or larval fixation, which reveals static algal presence and distribution, but prevents dynamic observation of the host and host-symbiont interactions^5^. This limitation represents a central challenge in marine biology; without the capability of live imaging, early symbiosis initiation and maintenance remains poorly understood and therefore difficult to address via new approaches (*e.g*. bioremediation for widespread coral bleaching). Live imaging capabilities would expand the toolkit for phenotypic analysis of this model organism and enable new avenues for assessing organism behavior under different environmental conditions. We therefore sought to develop an easy-to-use device for live, longitudinal observation of individual Aiptasia larvae and their algal symbionts over long time courses (2–12 hours) and under different environmental stressors as a model system for the study of cnidarian bleaching.

Microfluidic devices have served as excellent platforms for similar long-term imaging tasks in mammalian cells^14,15^, bacteria^16–18^, and plants^19,20^. Indeed, a ‘coral-on-a-chip’ microfluidic platform was previously developed for bulk reef-building coral microscopy^21^. However, this platform imaged non-motile juvenile coral that passively settled in the device and cannot be translated to the study of highly motile larvae. To our knowledge, no published study has demonstrated time-lapsed live imaging of Aiptasia larvae (or other similar cnidarian larvae) and their associated algal symbionts, likely due to the challenges associated with designing structures to immobilize deformable, highly motile cnidarian larvae that are large (∼90–140 *μ*m in length, ∼75–100 *μ*m in diameter) in size.

Here, we present a single-layer microfluidic device and associated hardware setup for isolating, trapping, and imaging up to 90 live individual Aiptasia larvae and their algal symbionts in parallel. We designed the device as part of a university microfluidics laboratory course that brought together engineers and life scientists to find solutions for pressing biological problems. The device is designed for operation by life scientists unfamiliar with microfluidics, requires only simple and inexpensive equipment, and is compatible with standard cnidarian-rearing protocols.

Optimizing the ‘Traptasia’ device required careful consideration of multiple trap geometries and organism sizes. To test how these parameters affect trap performance, we quantified trap occupancies for polymer beads of various sizes in devices of different heights and trap apertures. To ensure that adequate nutrients would reach trapped organisms, we simulated flow fields within and around occupied and unoccupied traps. The device is sized for a typical 1′ x 3′ coverslip and can easily be adapted for other medium- to large-sized (30–150 *μ*m in length, 30–150 *μ*m in diameter) motile organisms beyond the trapping capabilities of most mammalian cell hydrodynamic traps^14,15,22^. Using an optimized trap design for Aiptasia, we performed live imaging with Aiptasia larvae and their algal symbionts under constant flow and demonstrated the device’s potential for bleaching studies via capture of an algal expulsion event under the environmental stressor DCMU (3-(3,4-dichlorophenyl)-1,1-dimethylurea, known by its trade name Diuron). These experiments highlight the utility of the device for studying early cnidarian symbiosis and investigating the mechanisms of stress-driven cnidarian bleaching. Moreover, our modeling and bead-loading results provide critical information for future adaptation of these devices to study a wide range of large, deformable, and motile organisms currently underexplored in device literature.

## Results

### Microfluidic device design and experimental setup

A microfluidic device capable of studying anemone larval-algal symbiosis and mechanisms of algal expulsion must satisfy several design criteria. First, the device must stably trap deformable and highly motile Aiptasia larvae at medium- to high-throughput (10–50 larvae per experiment) over several hours (2–24 hours) while retaining organism viability. We selected this throughput to be competitive with larval fixation studies^13^, while additionally allowing longitudinal data collection from the same larva through time. Second, the device must allow for sufficiently high spatial and temporal resolution in both transmitted light and fluorescence channels (for imaging algal autofluorescence) to image larvae (∼90–140 *μ*m in length, ∼75–100 *μ*m in diameter), associated internal structures (*e.g*. gastrodermal layer and gastric cavity), and their algal symbionts (∼5–10 *μ*m in length). Finally, an ideal device for use in life science laboratories requires little setup and training time (∼30 and ∼15 minutes, respectively) and can be operated using common laboratory equipment (*e.g*. syringe pumps) with the ability to infuse fresh solutions and differential environmental treatments as desired for each experiment.

**Figure 1:**
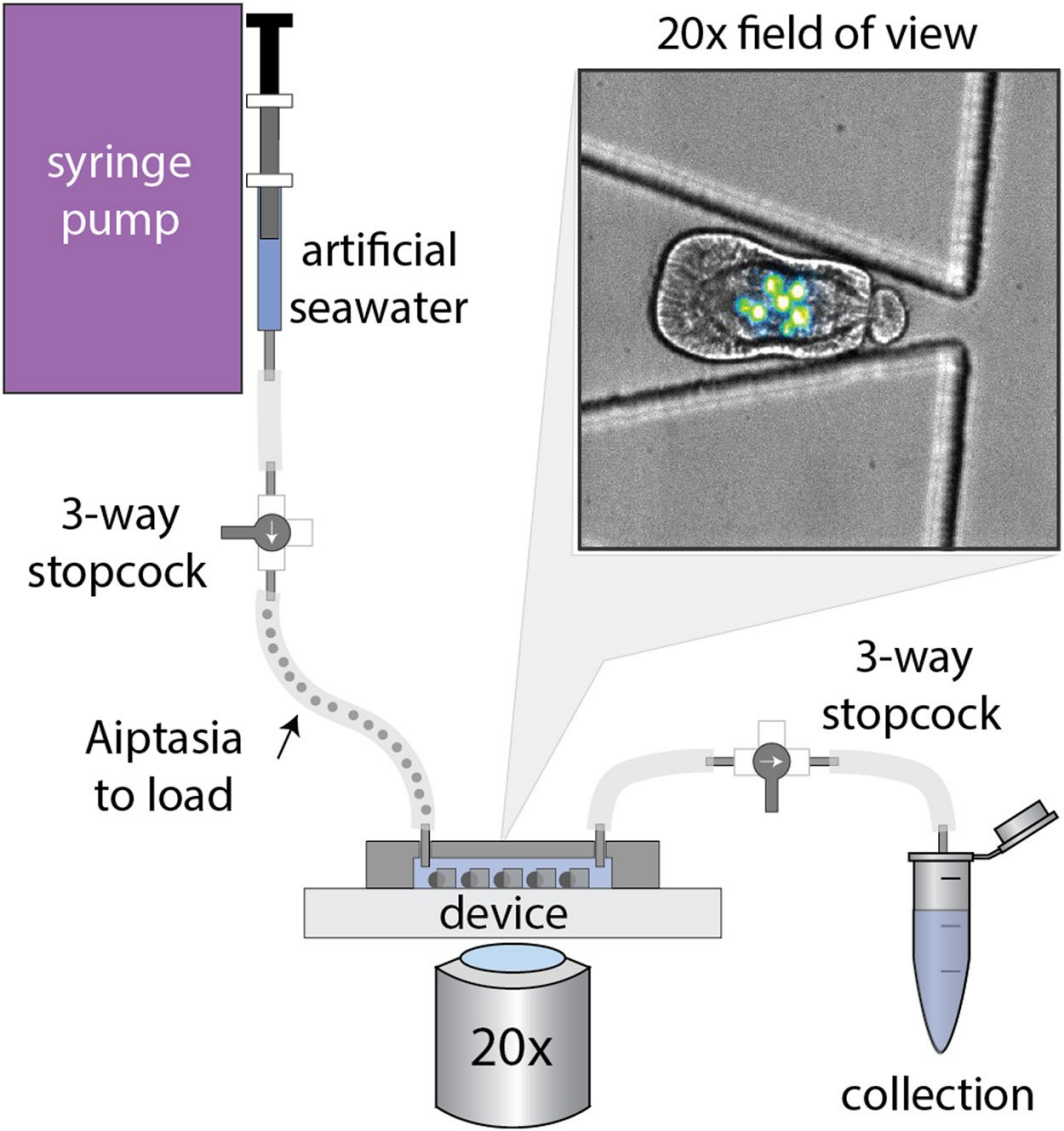
Schematic of the ‘Traptasia’ device and associated hardware for loading, trapping, and imaging individual Aiptasia larva. Organisms are loaded downstream of a stopcock assembly and connected to the device inlet. An upstream syringe pump provides constant fluid flow for trapping as well as nutrient and/or treatment delivery to the larva. Inset shows sample field of view with stably trapped Aiptasia larva (brightfield) and algae symbionts (blue-green, chlorophyll autofluorescence).

To meet these criteria, we designed a single-layer polydimethylsiloxane (PDMS) trapping device (termed ‘Traptasia’ for Trapping of Aiptasia) that requires only a syringe pump as necessary additional equipment and can be mounted on most common microscope setups (Fig. 1). The device is designed for ease-of-use and robust operation; organisms are loaded downstream of a stopcock assembly and connected to the microfluidic device. The inlet stopcock assembly is then connected to a syringe pump to load and exchange seawater, nutrients or drug treatments while minimizing bubbles or disruptions. When the syringe pump is turned on, organisms are loaded into the trapping array of the device and hydrodynamically trapped by constant fluid flow. Full details on operation and troubleshooting, including a step-by-step protocol, are available in *Supplemental Information*.

**Figure 2:**
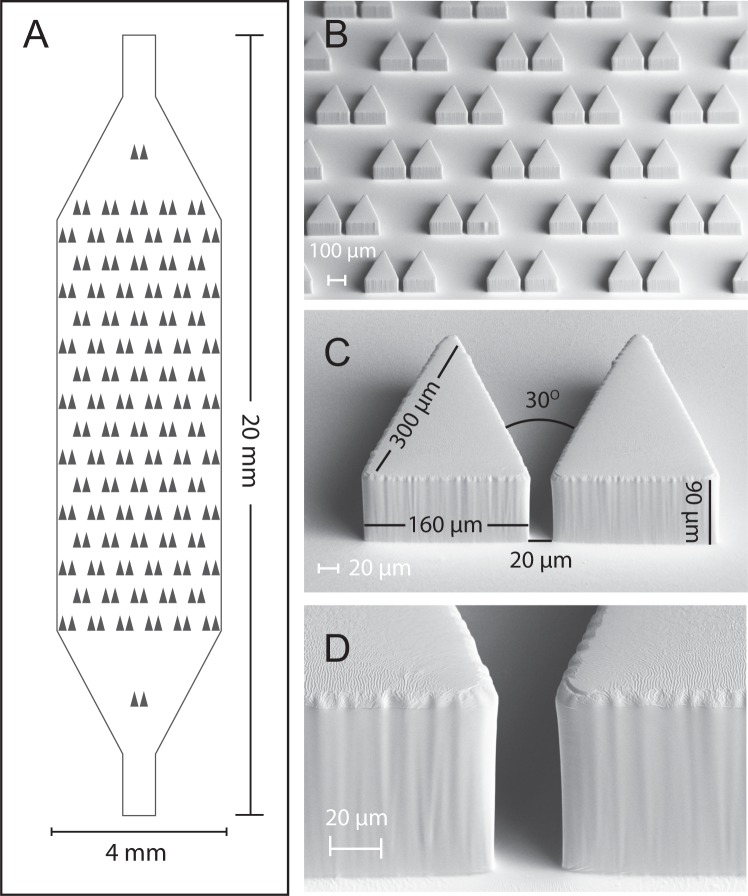
Single-layer microfluidic device for trapping individual Aiptasia larvae. (**A**) Design schematic for ‘Traptasia’ device containing an array of 90 triangular traps and tubing inlets. The final PDMS device is bonded to a No. 1 coverslip for imaging. (**B**) SEM image of PDMS device showing trap array. (**C**) SEM image of a single trap with labeled dimensions. (**D**) SEM image of trap aperture walls.

Each device contains an array of 90 paired isosceles triangle traps at 30° pitch (15° relative a normal line bisecting the pair) spanning the full chamber height (Fig. 2A–C); each triangle trap is 373 *μ*m long and 200 *μ*m wide with a variable trap aperture between triangles (Fig. 2B). Traps were laterally spaced by 280 *μ*m and arranged in 16 rows with alternating spacing to prevent clogs and promote flow between traps. Two additional traps were placed near the inlet and outlet of the device to prevent chamber collapse. The full trap array totals 20,000 *μ*m in length and 4,000 *μ*m in width with a single inlet and single outlet port, both designed for 23G pin to external tubing connections (*e.g*. Tygon). These devices are readily compatible with standard inverted microscopes in life science laboratories (Fig. S1).

### Validation of the ‘Traptasia’ array for large cell and organism loading

**Figure 3:**
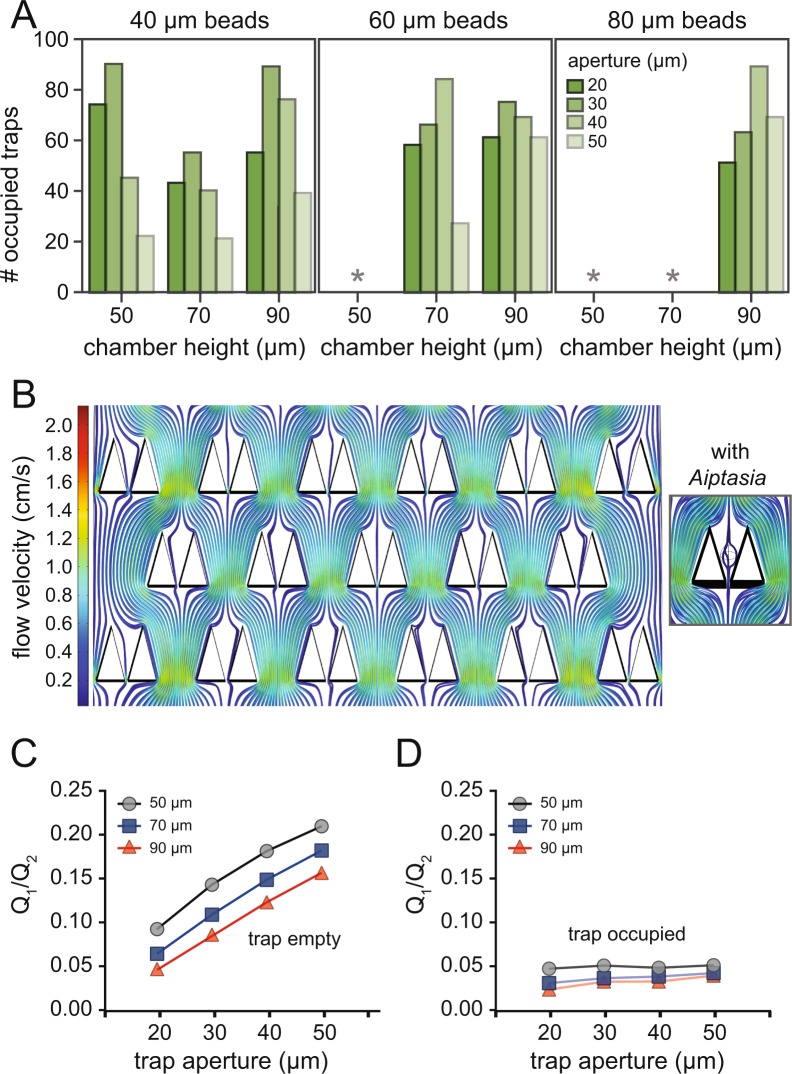
Trap-loading efficiencies and fluid flow simulations. (**A**) Bead occupancy histograms from bead loading experiments with different trap apertures, chamber heights and bead sizes. Gray asterisks denote instances when beads were too large to consistently enter chambers. (**B**) Simulated flow profiles within and between unoccupied traps under laminar flow conditions. Inset shows flow profiles around a rigid body as a proxy for an Aiptasia larva. (**C**,**D**) Ratio of simulated flow within and between traps (Q1/Q2) when unoccupied (**C**) and (**D**) occupied by simulated Aiptasia larvae for 3 chamber heights.

To enable medium- to high-throughput imaging of trapped organisms, specimens must be efficiently loaded in the device trapping array. To assess trap loading efficiencies, we systematically determined trap occupancy for different trap geometries using polymer beads as a proxy for organisms of different sizes. Polystyrene beads (∼5000 beads per condition, similar to projected specimen concentrations) of ∼40, 80, and 100 *μ*m mean diameters were loaded into ‘Traptasia’ devices with trap apertures of 20, 30, 40, and 50 *μ*m and chamber heights of 50, 70, and 90 *μ*m. Trap occupancy across the trapping array was determined after flow stabilization at 100 *μ*L/min (Fig. 3A). Beads larger than the chamber height were unable to reliably enter the device (as confirmed by imaging beads at the device inlet) and were excluded from further analysis (gray asterisks, (Fig. 3A)).

Aiptasia larvae are highly deformable, suggesting that geometries that efficiently trap beads over a wide size range (*e.g*., 40–100 *μ*m) would work best. Small beads (∼40 *μ*m) were trapped efficiently at all chamber heights at narrow trap apertures (20, 30, 40 *μ*m), but often escaped through or around traps with the largest aperture (50 *μ*m). This trend persisted for 60 and 80 *μ*m beads in taller devices (70, 90 *μ*m chambers), but narrow trap apertures (20, 30 *μ*m) yielded slightly lower occupancies. This likely results from increased fluidic resistance at lower trap apertures, as well as an observed higher propensity for clogging at device inlets. Significant challenges associated with loading and quantifying occupancy for large beads are noted in *Supplemental Information*, with suggestions that could prove useful for adapting these devices to other large non-model organisms.

From these data, we estimated that Aiptasia larvae of size range ∼40–100 *μ*m (under deformation) should occupy 20–80 of 90 potential traps per experiment when ∼5,000 larvae (a typical spawn size for larval culture) are loaded, which represents the full span of bead-loading occupancies. During bead-loading experiments, most traps were occupied within the first ‘burst’ of flow (∼10 s, <1000 beads), suggesting trap occupancy may not linearly increase with excess loading concentrations. Because of the observed deformability of Aiptasia larvae in dish culture and later in trapping experiments, as well as the variability in spawn sizes (ranging from 100–5,000 organisms per device loading), these rigid bead experiments at 5,000 beads per condition represent an upper bound for projected trap occupancy.

Using these bead-loading results as a guide, we tested loading live Aiptasia larvae into trap arrays with 90 *μ*m tall chambers and 20 and 30 *μ*m trap apertures. Unlike polymer beads, larvae are highly deformable; we found larvae could squeeze through 30 *μ*m apertures, but remained effectively trapped by 20 *μ*m apertures during preliminary testing. Differential bead counts at this geometry also suggested traps would predominately be occupied by a single organism (Fig. S2), an important parameter for ensuring each individual larvae would receive similar nutrient flow or drug treatment. We subsequently found these predictions of singlet occupancy to be correct in our live imaging studies.

### Simulations of flow profile around trapped aiptasia

Hydrodynamic trapping relies on maintenance of adequate volumetric flow through each Aiptasia larvae trap to prevent bubble nucleation or organism escape by swimming out of traps. Long-term (2–24 hour) imaging of Aiptasia larvae additionally requires that larvae receive adequate nutrient flow (in the form of oxygenated, artificial seawater) to maintain viability. Furthermore, if an environmental treatment (*e.g*. DCMU or another drug) is required for the experiment, treatments must be uniformly applied to each trap to minimize experimental variability.

Within the laminar flow regime in microfluidic devices, passive diffusion is minimal; fresh seawater, nutrients (if necessary), and treatments must be delivered via flow. To probe effects of trap occupancy on fluid flow, we simulated flow through unoccupied or Aiptasia-occupied traps (modeled as a rigid body) and calculated the relative fluid flow ratio (Q1/Q2) through (Q1) and around (Q2) traps at 100 *μ*L/min using COMSOL (Fig. 3B–D). All geometries had non-zero Q1/Q2 values, indicating sufficient flow through traps for seawater and treatment delivery and suggesting differential hydrodynamic trapping. In particular, the 90 *μ*m chamber height with a 20 *μ*m trap aperture resulted in a Q1/Q2 = 0.046 for an unoccupied trap and Q1/Q2 = 0.025 for a rigid ellipsoid-occupied trap. The latter creates a lower bound for fluid flow in the case of a highly non-deformable cell type; Aiptasia are likely to experience increased flow due to flow-induced deformation.

We next simulated flow profiles throughout a device with 50% occupied traps, distributed randomly. Flow through these traps (occupied or vacant) at a volumetric flow of 100 *μ*L/min ranged between 0.0132 and 0.005 *μ*L/s. Even at this lower bound, traps would receive 1 Aiptasia-volume worth of flow (0.0006 *μ*L) in less than 1 second, sufficient for long-term incubation in the device. These simulations also revealed that flow rates were relatively uniform for different traps in the array even under randomly distributed occupancy (Fig. S3), an important consideration for drug or environmental treatments to multiple larvae in the same device. Taken together, these data establish that fresh seawater and any desired treatment should reach and sustain trapped organisms.

### ‘Traptasia’ Enables Live Imaging of Aiptasia Larvae and Algal Symbionts for Long Time Courses

Longitudinal measurements of single trapped Aiptasia can reveal organism behavior and symbiosis dynamics that snapshots of fixed larvae cannot. To perform long time-course imaging of trapped larvae, we mounted the ‘Traptasia’ microfluidic device on a spinning disk confocal microscope. We loaded larvae co-infected with algae into the device and imaged each occupied trap in the array at 40 minute intervals and at multiple heights (3 *μ*m stepped z-stacks) under a constant seawater flow to monitor the larval-algal symbiotic relationship.

Brightfield images of larvae clearly resolved animal margins and internal larval anatomy including the gastroderm, gastric cavity boundaries, cilia, and the apical tuft (Fig. S4). Simultaneously acquired fluorescence images in a custom far-red fluorescence channel corresponding to chlorophyll autofluorescence allowed visualization of algal symbionts within the Aiptasia larval gastroderm (false colored to blue-green, Fig. S4). Currently, to our knowledge, no dyes exist and/or are capable of membrane penetration for live-organism staining for anatomical borders (*e.g*. phalloidin for actin boundaries requires fixation) but if these dyes become available, they would be easy to integrate in tandem with brightfield imaging similar to the chlorophyll autofluorescence data presented here.

**Figure 4:**
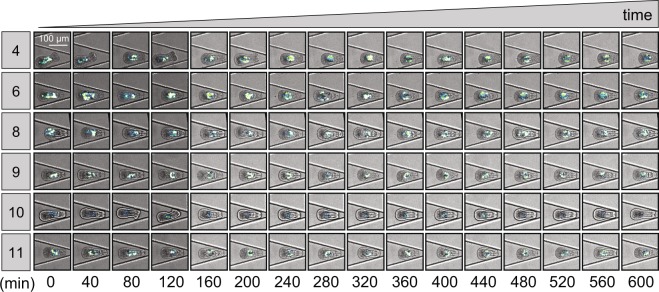
Time course of select traps demonstrating stable trapping of viable Aiptasia larvae for 10 hours. Each row represents a single trap (TrapID, left); each column represents an imaged time point. Each image is a merged brightfield and fluorescence image to show device trap, trapped larva, and algal symbionts. All traps shown in (Figs S5 and S6).

In multiple experiments, we obtained images of >100 individual Aiptasia larvae, demonstrating stable trapping within the ‘Traptasia’ array (further details described in Device Operation note in *Supplemental Information*). In one experiment, 25 of 90 traps remained occupied by larvae (each with 5–30 algal symbionts) under 35 *μ*L/min seawater flow for >10 hours (example traps, Fig. 4). Of these larvae, all 25 remained viable for the duration of the 10 hour experiment (Figs S5 and S6). After flow was terminated at. ∼11.5 hours, 8 larvae swam out of the trap (TrapIDs: 23–32, 35), and 1 underwent lysis (TrapID: 26) (Fig. S7).

### Detailed algal symbiont imaging in individual larvae

The ability to visualize and track individual algal symbionts within live larvae during homeostasis and under stress provides a new way to identify the mechanisms of cnidarian-algal symbiosis. In addition, capturing algal expulsion events may provide insight into related reef bleaching. Current algal-tracking methods require chemical fixation of Aiptasia before confocal or electron microscopy and thus cannot provide dynamic information about algal movements^5^. Trapped Aiptasia larvae were viable with free-moving cilia in ‘Traptasia’ devices with 90 *μ*m chamber heights but appeared slightly compressed when viewed throughout the entire z-stack of imaging, suggesting that the devices could stabilize motile larvae for algal symbiont imaging (explemary z-stack, Movie S2).

**Figure 5:**
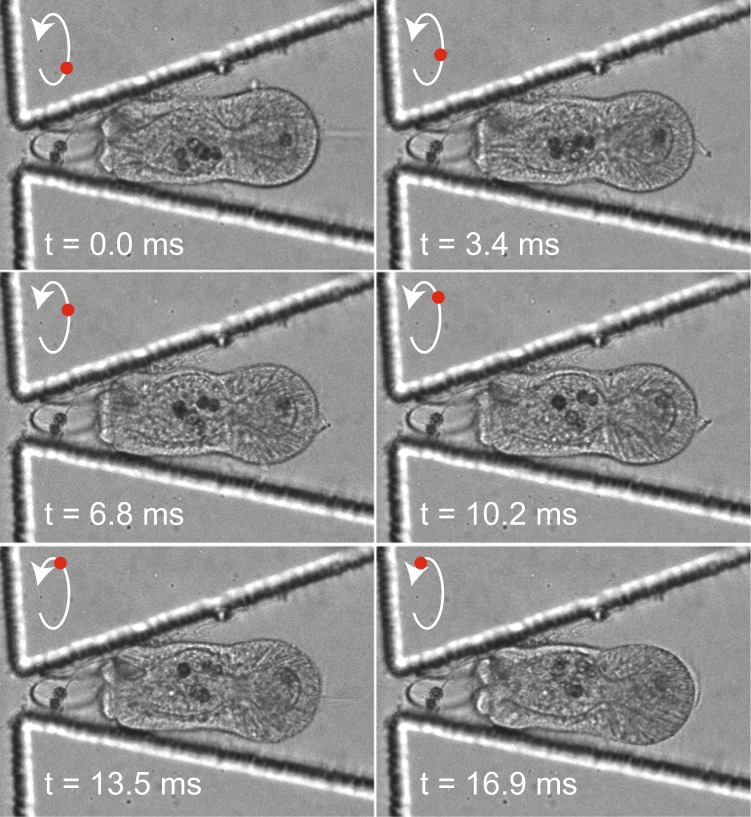
Time subset of brightfield imaging of a trapped Aiptasia larva at high temporal resolution demonstrating the ability to resolve algal movement and monitor larval revolutions (larval spin, relative to lengthwise normal, denoted by white arrow; estimated position in revolution denoted by red dot) within the trap. Frames were extracted at a single plane every 5 time steps (for visualization) from a continuous time-lapse sequence. Full image sequence can be visualized in Movie S3.

To track individual algae, we trapped larvae co-infected with algal symbionts and performed fast-acquisition imaging in transmitted light alone (50 ms exposures, 5 × 3 *μ*m z-stacks) to obtain continuous time series for selected individual larvae. Under normal seawater conditions at 100 *μ*L/min flow, algae appeared to freely move within the gastroderm, driven mostly by larval spin, without entering the gastric cavity (Fig. 5). Algae counts per organism, resolved through brightfield imaging, remained constant throughout acquisition (Movies S3 and S4 as examples). Larval revolutions, including change in direction and speed, were clearly visible, providing additional opportunities to monitor complex or subtle stress responses (Movies S3 and S4). The ability to resolve individual algae within larvae without confocal microscopy could allow use of the device within a broad range of environments (*e.g*. undergraduate research institutions, local museums or aquaria, or field research stations).

### Multiple larval-death mechanisms revealed by imaging

Viable Aiptasia larvae show robust ciliary movement and low-speed rotation. During our trials, we observed multiple events of larval death characterized by high rotation within traps, lack of ciliary movement, or cellular degradation (Fig. S8). Bubble nucleation within the microfluidic device during initial trials often resulted in larval death (Fig. S8c), but was mitigated entirely with 100 *μ*L/min flow speeds in combination with device debubbling (see *Supplemental Information* and our Open Science Framework for full imaging datasets).

Beyond device-related larval death phenotypes, we observed interesting, potentially physiologically-relevant instances of organism degradation. In some cases, dying larvae separated their mouth from their gastric cavity prior to complete degradation (Movie S5), a phenomenon that has also been observed in our larval cultures. In other cnidarian species, mouth separation may precede opening for feeding or organism regeneration^23^, but these mouth separation events merit further study in Aiptasia to determine physiological significance. In other cases, larval degradation appeared to result from ciliate infestation, likely acquired during algal infection (Fig. S8b). These events are frequently observed in bulk culture but have never been visualized in cellular detail, illustrating how the ‘Traptasia’ device can facilitate detailed investigation of multiple aspects of cnidarian physiology.

### Observation of algal symbiont expulsion from a larval host under a proposed environmental stressor

To investigate the cellular mechanisms that drive cnidarian bleaching, we introduced a proposed coral stressor, DCMU (3-(3,4-dichlorophenyl)-1,1-dimethylurea, trade name Diuron) to trapped Aiptasia larvae and their algal symbionts. DCMU is a potent inhibitor of photosystem II, thereby disrupting photosynthetic pathways in algae and, thus, causing metabolic harm to both algal symbionts and their host^24^. DCMU has high environmental relevance for the study of cnidarian bleaching; it is a herbicide introduced into the marine environment from agricultural runoff and antifouling paints^24^ and routinely used for physiological induction of algal bleaching in Aiptasia and other anemones and corals^12,24–26^. Consistent with prior work^12,25,26^, we introduced DCMU at 25 *μ*M concentration to trapped larvae to investigate the mechanisms of algal expulsion by induction within our device. This concentration may represent an upper bound of environmental relevance, and future work across wider DCMU concentration ranges could prove useful for a furthered understanding of environmental toxicity limits.

In the presence of 25 *μ*M DCMU in seawater at 35 *μ*L/min flow rates, 33 Aiptasia larvae and their algal symbionts were stably trapped within 90 possible trapping positions (Fig. S9). These data directly compare to those presented in the seawater alone experiments (Figs S5 and S6). Of the 33 trapped larvae, 28 remained trapped for the course of the imaging acquisition (80 minutes). Five larvae ‘swam’ upstream or through the trap aperture (Trap IDs: 8, 10, 11, 29, 42), suggesting that larval motility and deformation may increase under stress as compared to seawater.

**Figure 6:**
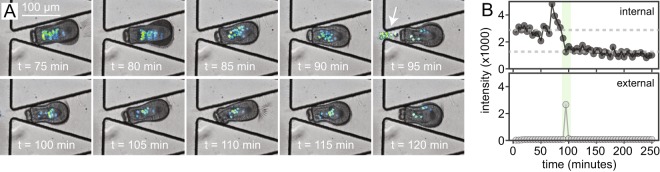
Algal expulsion event from a live larva. (**A**) Time-lapse imaging sequence showing clear evidence of an algal expulsion event from the larval mouth through the gastric cavity (white arrow, 95 minutes). (**B**) Mean measured fluorescent intensity (photon counts) for a equal-size region of interest within (top panel) and external to (bottom panel) Aiptasia boundaries (sum intensity across the z-projection of the z-stack for each timepoint). Fluorescence within Aiptasia decreases by about one third at the same frame that expelled algae are visible outside the larval body.

Under DCMU treatment, we captured an algal expulsion event from a live Aiptasia larva (Fig. 6A, Movie S6). Quantification of mean algal autofluorescence within larval margins and the surrounding external environment from sum projection (summed intensity of the fluorescence channel through the entire z-stack) before, during, and after the expulsion of algal symbionts from the larval gastric cavity and mouth reveals a strong signal gain during expulsion in the external environment and consequent loss within the host margins. This analysis provides a high-throughput method for identifying expulsion events without manual inspection (Fig. 6B). To our knowledge, these data represent the first dynamic visualization of an algal expulsion event, thought to be the critical event underlying widespread cnidarian (coral) bleaching. In future implementations of the device, systematic trials of larval imaging under different stressors could capture more expulsion events and further elucidate biological mechanisms behind larval stress induction and algal expulsion.

## Discussion

In this report, we developed an easy-to-operate microfluidic device and setup (‘Traptasia’) for trapping and imaging sea anemone (Aiptasia) larvae and their algal symbionts over long time courses. This device addresses a long-standing limitation in the ability to image live, highly motile larvae for mechanistic studies of the processes that drive cnidarian bleaching. We demonstrate the utility of the device both in high-throughput imaging and detailed time-lapse imaging tasks. While simple, our device operates robustly and reliably and can easily be adapted to other large, understudied cell types or microorganisms. We believe this device will provide a new capability to researchers, allowing them to produce longitudinal data on large numbers of live Aiptasia experiencing different chemical and environmental stresses, previously inaccessible at any throughput.

In confirming the viability of the ‘Traptasia’ device design, we conducted bead occupancy tests across a wide array of trap geometries with trap apertures of [20, 30, 40, 50] *μ*m and chamber heights of [50, 75, 90] *μ*m. These occupancy experiments were used to predict device geometries for successful Aiptasia trapping, but they could easily be used to estimate optimal trap geometries and occupancies for other large and highly motile organisms. While prior data exist for mammalian single cell traps, such data for large (>30 *μ*m size) organisms are not readily available.

Similarly, our flow field calculations across different device geometries also have utility outside these studies. In future work, these flow profiles will help inform environmental stressor concentration dosing and resultant effective agent concentrations experienced by each Aiptasia larva. Additionally, this flow modeling approach may help inform nutrient concentration requirements for organisms requiring more complex nutrient solution delivery to sustain long-term study.

In the future, this device could facilitate the study of Aiptasia-algal symbiosis under healthy conditions and in the presence of known and proposed inducers of coral bleaching (*e.g*. increased temperature, increased salinity, DCMU and other chemicals). The device could also help answer basic mechanistic biological questions about the stability of algal symbiosis, the frequency of healthy vs. non-healthy expulsion events, and organism-to-organism heterogeneity in symbiotic behavior (i.e., migration and algal movement). Combined with reverse-flow techniques for retrieving the larvae post-analysis, additional downstream assays such as genomic^8^ or transcriptomic^7,9^ profiling could help identify algal-stress mechanisms.

This device continues to be used for mechanistic biological studies of cnidarian-dinoflagellate symbiosis in collaborating laboratories at Stanford and the Carnegie Institute for Science. In future work, we hope the device can be adopted more broadly, both for experiments involving sea anemones, corals and other cnidarians, as well as other large, motile organisms. By sharing these data, design files, and detailed protocols, we hope that other biologists and researchers will adopt this, or similar, trapping devices for their own research.

## Methods

### Animal culture and maintenance

Small sea anemone (Aiptasia) larvae were cultured in the laboratory^12^. Larvae were obtained via induced spawning of clonal adult anemone lines CC7 (male) and PLF3 (female) in paired co-culture in artificial sea water (ASW; 34 ppt Coral Pro Salt in deionized (DI) water, pH 8.0, Red Sea Inc.)^9^. To set up anemones for spawning, adults from each clonal population were selected by size (>15 mm oral disk size) and manually paired (single male to single female) in polycarbonate tanks of 300 mL of ASW to induce spawning under 27 °C incubation (Percival). Co-cultures were maintained on a 12-hour light: 12-hour dark cycle with 25 Âµmol photons/(m^2^ s) white fluorescent light (Philips ALTO II 25W) (light cycle) for 25 days and subsequently switched to a 16-hour light: 8-hour dark cycle for 5 days, with an additional 1 Âµmol photons/(m^2^ s) actinic blue LED (Current USA TrueLumen) during the dark cycle. One-day-old larvae were re-suspended in 1 mL ASW in wells of a 24-well polypropylene (PPE) plate at a density of 1,000–3,000 larvae per well before infection with algae.

### Algal infection for symbiosis studies

Freshly isolated algae were used to infect Aiptasia larvae in this study. *Symbiodinium* (clade A) were isolated from an individual CC7 adult anemone by homogenization (PowerGen 125 rotor, Fisher Scientific) at 30,000 rpm for 10 s, restrictive mixing via a 25G needle attached to a 1 mL syringe (BD Biosciences) at 5 repetitions and centrifugation at 1000 × g for 2 min to pellet algal cells. Intact algal cell pellets were washed 10 times in fresh ASW and re-suspended 1:1 v/v in wells of the 24-well PPE plate containing larvae. The plate was maintained on a 12-hour light: 12-hour dark cycle at 27 °C prior to use in imaging studies in the microfluidic device platform within 1–2 days of infection. Larvae were 3–5 days old at the start of the experiment.

### Device design, photolithography, and fabrication

Devices of trap apertures of [20, 30, 40, 50] *μ*m and chamber heights of [50, 70, 90] *μ*m were designed and fabricated for bead loading and empiric optimization trials; 90 *μ*m chamber height with 20 *μ*m aperture were used for all subsequent Aiptasia experiments. All devices were fabricated via standard soft lithography protocols^27^. Design files used in this study are provided as Supplemental Resource 1. Full lithography details are provided in *Supplemental Information*, (Table S3) and (Fig. S10).

### Bead-Loading experiments

Different sizes of polystyrene cross-linked polymer beads (40, 60, 80 *μ*m general distribution, products PPX-400, PPX-600, PPX-800, Spherotech) were assessed in the ‘Traptasia’ device. Using a similar setup to that used for Aiptasia experiments, per each condition ∼5000 beads at constant volume were loaded for ∼10 s, the device was subsequently de-bubbled, and loading was continued and allowed to stabilize for 2 minutes under 100 *μ*L/min flow (infuse-only) using a syringe pump (Pico Pump, Harvard Apparatus).

### COMSOL fluid modeling

Fluid flow simulations to assess flow fields and model nutrient flow through traps with and without Aiptasia were modeled in COMSOL (COMSOL Multiphysics) using the 3D Laminar Flow module. Inlet velocity was set at 100 *μ*L/min and outlet pressure was set to 1 atm. Aiptasia-occupied traps were simulated by placing a rigid ellipsoid of volume $5.9\ast {10}^{5}$ *μ*m^3^ 1 *μ*m away from the trap aperture center for trapped flow field analysis. Further parameters are described in (Table S-T1).

### SEM imaging

SEM Images of the device were acquired using a Zeiss Sigma Scanning Electron Microscope (SEM) with Schottky Field Emission (FE) source and GEMINI electron optical column at the Stanford Microscopy Facility. The accelerating voltage was set by magnification: 4.0 kV at 500–1,700X for imaging individual traps and apertures, 10.0 kV at 50X for imaging the full trap array width. Devices were sputter-coated with 10 nm of gold-palladium and imaged at 60° tilt.

### Device loading of aiptasia larvae

To perform Aiptasia imaging experiments, Aiptasia larvae were loaded directly from culture wells (∼100 *μ*L) using 1/16″ tubing (Tygon, Fisher) attached to a 1 mL syringe (Plastipak, BD) with a 23 G luer-lock connector (McMaster-Carr). Lines were hung from the syringe pump vertically for 2 minutes to concentrate the motile larvae and connected upstream of the inlet stopcock. A step-by-step loading and operation protocol relative to each trial is provided in *Supplemental Information*. In trials with DCMU treatment, seawater containing 25 *μ*M DCMU (Diuron, Sigma) was introduced instead of normal seawater after larval loading.

### Image acquisition

Four-dimensional imaging data (time, fluorescence channels, and z-slices) of Aiptasia larvae in the microfluidic device traps were acquired on a Leica DMI6000B stand equipped with a Yokogawa CSU-10 spinning disk head, QuantEM camera (Photometrics), and ASI MS2000 motorized XY stage under 20X magnification (Leica 20X/0.7 NA multi-immersion, used with glycerine solution). Z-stacks were collected in transmitted light and chlorophyll autofluorescence channels (561 nm excitation, 405/488/561 nm dichroic and 637/37 nm emission, Semrock), as noted, under control of SlideBook 6 (Intelligent Imaging Innovations).

### Image analysis

Images were analyzed in Fiji (ImageJ)^28^. For algal fluorescence quantification, images in the time series were z-projected from the z-stack by sum intensity on the chlorophyll auto-fluorescence channel and minimal intensity on transmitted light; data in Fig. 6B is generated from mean fluorescence values (arb. units) extracted from equal size ROIs in the animal, expulsion area, and background per frame from sum projection image in the algal fluorescence channel for each frame. For simple visualization, mid-plane z-slices in both channels were displayed and false-colored by transmitted light (grayscale) and chlorophyll autofluorescence (Lookup Table: Green Fire Blue) to create time-course images and movies.

## Supplementary information

Movie S1

Movie S2

Movie S3

Movie S4

Movie S5

Movie S6

Supplementary Information

## References

[1] Hoegh-Guldberg O (2007). Coral Reefs Under Rapid Climate Change and Ocean Acidification. Science.

[2] Davy SK, Allemand D, Weis VM (2012). Cell biology of cnidarian-dinoflagellate symbiosis. Microbiology and Molecular Biology Reviews.

[3] Weis VM, Davy SK, Hoegh-Guldberg O, Rodriguez-Lanetty M, Pringle JR (2008). Cell biology in model systems as the key to understanding corals. Trends in Ecology & Evolution.

[4] Yellowlees D, Rees TAV, Leggat W (2008). Metabolic interactions between algal symbionts and invertebrate hosts. Plant, Cell & Environment.

[5] Bieri T, Onishi M, Xiang T, Grossman AR, Pringle JR (2016). Relative Contributions of Various Cellular Mechanisms to Loss of Algae during Cnidarian Bleaching. PLoS ONE.

[6] Bucher M, Wolfowicz I, Voss PA, Hambleton EA, Guse A (2016). Development and Symbiosis Establishment in the Cnidarian Endosymbiosis Model *Aiptasia sp*. Scientific Reports.

[7] Lehnert EM, Burriesci MS, Pringle JR (2012). Developing the anemone *Aiptasia* as a tractable model for cnidarian-dinoflagellate symbiosis: the transcriptome of aposymbiotic *A. pallida*. BMC Genomics.

[8] Baumgarten S (2015). The genome of *Aiptasia*, a sea anemone model for coral symbiosis. Proceedings of the National Academy of Sciences.

[9] Sunagawa S (2009). Generation and analysis of transcriptomic resources for a model system on the rise: the sea anemone *Aiptasia pallida* and its dinoflagellate endosymbiont. BMC Genomics.

[10] Cumbo VR, Baird AH, van Oppen MJH (2012). The promiscuous larvae: flexibility in the establishment of symbiosis in corals. Coral Reefs.

[11] Wolfowicz I (2016). *Aiptasia sp*. larvae as a model to reveal mechanisms of symbiont selection in cnidarians. Scientific Reports.

[12] Xiang Tingting, Jinkerson Robert E., Clowez Sophie, Tran Cawa, Krediet Cory J., Onishi Masayuki, Cleves Phillip A., Pringle John R., Grossman Arthur R. (2017). Glucose-Induced Trophic Shift in an Endosymbiont Dinoflagellate with Physiological and Molecular Consequences. Plant Physiology.

[13] Hambleton EA, Guse A, Pringle JR (2014). Similar specificities of symbiont uptake by adults and larvae in an anemone model system for coral biology. Journal of Experimental Biology.

[14] Khademhosseini A (2005). Cell docking inside microwells within reversibly sealed microfluidic channels for fabricating multiphenotype cell arrays. Lab on a Chip.

[15] Cui X (2017). A microfluidic device for isolation and characterization of transendothelial migrating cancer cells. Biomicrofluidics.

[16] Sachs CC (2016). Image-Based Single Cell Profiling: High-Throughput Processing of Mother Machine Experiments. PLoS ONE.

[17] Kaiser M (2018). Monitoring single-cell gene regulation under dynamically controllable conditions with integrated microfluidics and software. Nature Communications.

[18] Bamford RA (2017). Investigating the physiology of viable but non-culturable bacteria by microfluidics and time-lapse microscopy. BMC biology.

[19] Massalha H, Korenblum E, Malitsky S, Shapiro OH, Aharoni A (2017). Live imaging of root–bacteria interactions in a microfluidics setup. Proceedings of the National Academy of Sciences.

[20] Grossmann G (2011). The RootChip: an integrated microfluidic chip for plant science. The Plant cell.

[21] Shapiro OH, Kramarsky-Winter E, Gavish AR, Stocker R, Vardi A (2016). A coral-on-a-chip microfluidic platform enabling live-imaging microscopy of reef-building corals. Nature Communications.

[22] Di Carlo D, Aghdam N, Lee LP (2006). Single-cell enzyme concentrations, kinetics, and inhibition analysis using high-density hydrodynamic cell isolation arrays. Analytical Chemistry.

[23] Carter JA, Hyland C, Steele RE, Collins E-MS (2016). Dynamics of Mouth Opening in Hydra. Biophysical journal.

[24] Jones R, Mueller JF, Haynes D, Schreider U (2003). Effects of herbicides diuron and atrazine on corals of the Great Barrier Reef, Australia. MEPS.

[25] Chakravarti, L. J., Negri, A. P. & Van Oppen, M. A. J. Thermal and Herbicide Tolerances of Chromerid Algae and Their Ability to Form a Symbiosis With Corals. *Frontiers in Microbiology* (2019). 10.3389/fmicb.2019.00173 PMC6379472 30809207

[26] Xiang T, Hambleton EA, DeNofrio JC, Pringle JR, Grossman AR (2013). Isolation of clonal axenic strains of the symbiotic dinoflagellate symbiodinum and their growth and host specificity. Journal of Phycology.

[27] Brower, K., White, A. K. & Fordyce, P. M. Multi-step Variable Height Photolithography for Valved Multilayer Microfluidic Devices. *Journal of Visualized Experiments* 1–12 (2017). 10.3791/55276 PMC5352304 28190039

[28] Schindelin J (2012). Fiji: an open-source platform for biological-image analysis. Nature Methods.

