# Supplementary material for: Live imaging of Aiptasia larvae, a model system for coral and anemone bleaching, using a simple microfluidic device
Source: Sci Rep. 2019 Jun 25;9:9275. doi: 10.1038/s41598-019-45167-2 (PMC6592900; doi:10.1038/s41598-019-45167-2)
Supplement: Supplementary file 7 — Supplementary Information [file 41598_2019_45167_MOESM7_ESM.pdf]

Supplementary Information for

Live imaging of Aiptasia larvae, a model system for coral and anemone bleaching, using a simple microfluidic device

Will Van Treuren*, Kara K. Brower*, Louai Labanieh, Daniel Hunt, Sarah Lensch, Bianca Cruz, Heather N. Cartwright, Cawa Tran, and Polly M. Fordyce

Polly Fordyce
E-mail: pfordyce@stanford.edu

This PDF file includes:

Supplementary text
Figs. S1 to S10
Tables S1 to S3
Captions for Movies S1 to S6
References for SI reference citations

Other supplementary materials for this manuscript include the following:

Movies S1 to S6

Supporting Information Text

Extended Methods

Device Design and Mask Printing. Devices of trap apertures of [20, 30, 40, 50] µm and chamber heights of [50, 70, 90] µm were designed and fabricated for bead loading and empiric optimization trials; 90 µm chamber height with 20 µm aperture were used for all subsequent Aiptasia experiments. All designs were generated in AutoCAD (Autodesk). Final triangle trap designs are available as **Supplemental Resource 1** and all designs used in the study at all stages of iteration are available on an **Open Science Framework** project for this work. Designs were printed on 32,000 DPI transparency film (Fineline Plotting) for subsequent photolithography and device fabrication.

Photolithography. Devices were fabricated via standard soft lithography protocols(1). Briefly, single-layer devices were prepared via photolithography and subsequent replica molding into PDMS negative relief channels in the Stanford Microfluidic Foundry. For photolithography, Si 4" test-grade wafers (University Wafer) were cleaned with methanol and dried for 10 minutes before use. A 5-µm adhesion layer was applied using SU-8 2005 (MicroChem Corp.), flood exposed and post-exposure baked at 95°C for 5 min. Subsequently, SU-8 photoresist (MicroChem Corp.) of the appropriate height was spin-coated, soft baked, exposed through the trap array transparency mask, post-exposure baked, developed and hard-baked according to the manufacturer's instructions. Full lithography details, including a detailed step-by-step instruction set, are provided in [**Table S-T3**] and [**Fig. S10**].

Device Fabrication. Post-hard-bake, wafers were silanized with aerosolized trichloro(1H, 1H, 2H, 2H-perfluorooctyl)silane, 97% (PFOTS, Sigma) for 10 minutes to aid PDMS transfer. PDMS (RTV 615, Momentive) was prepared according to manufacturer's instructions in a 1:10 ratio and 55 g was poured onto the master mold. The wafer was baked for 45 minutes at 80°C. Devices were peeled off, cut, and hole-punched for 23G pin-to-tubing connections. Devices were subsequently corona-wand treated for 30 s and aligned to No. 1 coverslips (Fisher Scientific) before being baked at 80°C for 12 hours to ensure strong bonding to the coverslip surface.

Device Operation Setup. Devices were connected in-line with a syringe pump and placed on a microscope slide mount before organism loading and imaging. The complete setup consists of:

1. a syringe pump (Pico-Plus, Harvard Apparatus) with a syringe (60 mL Plastipak, BD) and 23G luer connector (McMaster Carr) for loading larvae and seawater;

2. a luer-adapter one-way stopcock (Cole Parmer) with intermediary tubing to 1/16" tubing (Tygon, Fisher) connections for transferring inputs;

3. 1/16" tubing to PEEK HPLC tubing (510 µm O.D., 255 µm I.D., Zeus) for the device input line;

4. 23G steel pin (New England Small Tube) to 1/16" tubing device outlet connection; and

5. similar to the inlet stopcock assembly, a luer-adapter one-way stopcock (Cole Parmer) with intermediary tubing to 1/16" tubing (Tygon, Fisher) outlet connection to de-bubble the device and collect waste.

Optionally, an off-chip bubble trap (Elveflow) for eliminating in-line bubbles connected directly to the chip inlet via additional 1/16" tubing (Tygon, Fisher) and PEEK HPLC tubing (510 µm O.D., 255 µm I.D., Zeuss) can be included for further bubble control at flow rates below 100 µL/min. A parts list is available in [**Table S2**].

To change buffers, load cells, or add reagents, the syringe pump was stopped, the stopcock closed and tubing disconnected upstream and syringes were exchanged. This process minimized bubble introduction into the chip. A schematic of the device setup is included in [**Fig. S1**].

Preparation of the Microfluidic Device. To prevent bubbles common in long-term imaging studies using single-layer microfluidic devices, devices were surface-treated to render natively hydrophobic PDMS channel walls hydrophilic shortly before each experimental run. Devices were passivated with 5% BSA solution (BSA powder, Sigma) for 15 minutes at 35 µL/min flow using a syringe pump (Harvard Apparatus) to improve hydrophilicity and device wettability for studies conducted at low flow rates (35 µL/min); for later trials at high flow rates (100 µL/min, superior bubble mitigation), this step was unnecessary for bubble prevention. To de-bubble devices, the device outlet one-way stopcock (Cole Palmer) was turned off and the device was dead-end filled using the syringe pump at 100 µL/min flow under visual inspection until all bubbles were removed from the trap area (approximately 2 minutes). For Aiptasia runs, the syringe was exchanged to artificial seawater (ASW) and flushed through the device for 1 minute at 100 µL/min after de-bubbling to prepare for cell loading.

Bead-Loading Experiments, Extended. Bead-loading experiments were conducted across different device parameters including trap apertures of [20, 30, 40, 50] µm and chamber heights of [50, 70, 90] µm. Different sizes of polystyrene cross-linked polymer beads ([40, 60, 80] µm general distribution, products [PPX-400, PPX-600, PPX-800], Spherotech) were loaded into the 'Traptasia' device for each chamber and trap aperture combination. For each condition, ~5000 beads were loaded into a constant volume 1/16" Tygon to PEEK tubing assembly similar to later cell experiments and loaded into the chamber under 100

µL/min flow (infuse-only) using a syringe pump (Pico Pump, Harvard Apparatus). Bead amounts per sample were drawn from normalized dilutions of 100,000 beads/mL, estimated using manufacturer specifications and confirmed via a hemocytometer for each bead size. Using a similar setup to that used for Aiptasia experiments, beads were loaded for ~10 s, the device was subsequently de-bubbled, and loading was continued and allowed to stabilize for 2 minutes. In some instances (especially larger bead sizes), bead clogging was observed (see *Supplemental Extended Notes*) at the inlet region due to small hole punch sizes; such clogging was partially mitigated by opening and closing the outlet stopcock before flow stabilization. The microfluidic device was then imaged under brightfield illumination using an Amscope Stereoscope with an ZWO ASI-174MM camera at 10X magnification. Images were analyzed for bead occupancy in ImageJ using manual counting. Trapping efficiency was assessed by number of traps occupied per bead size and per device geometry as compared to total number of traps in the area (n=90 traps). Differential trap occupancy (number of beads per trap) was also assessed per each condition.

COMSOL Fluid Modeling, Extended. Fluid flow simulations to assess flow fields and model nutrient flow through traps with and without Aiptasia were modeled in COMSOL (COMSOL Multiphysics) using the 3D Laminar Flow module. Fluid flow through models with trap apertures of [20, 30, 40, 50] µm and chamber heights of [50, 70, 90] µm was assessed under assumptions of laminar flow. Inlet velocity was set at 100 µL/min and outlet pressure was set to 1 atm. All simulations are available under the **Open Science Framework** for this project. Aiptasia-occupied traps were simulated by placing a rigid ellipsoid of size $5.9 * 10^5$ μm^3 1 µm away from the trap aperture center for trapped flow field analysis. For smaller chamber heights, the rigid ellipsoid was scaled such that volume was conserved (to simulate deformed Aiptasia). Further parameters are described in [**Table S1**]. Flow rates were extracted from the sparse simulation at the half-width trap of trap aperture (Q1) and half-width between traps (Q2) for the comparative flow calculations.

Device Loading of Aiptasia Larvae. To perform Aiptasia imaging experiments, Aiptasia larvae were loaded directly from culture wells (~100 µL) using 1/16" tubing (Tygon, Fisher) attached to a 1 mL syringe (Plastipak, BD) with a 23G luer-lock connector (McMaster-Carr). Lines were hung from the syringe pump vertically for 2 minutes to concentrate the motile larvae, then connected upstream of the inlet stopcock or, in early trials, directly to the device. A full protocol is described later in *Supplemental Information.* In trials with DCMU treatment, seawater containing 25 µM DCMU (Diuron, Sigma), an herbicide and environmental stressor, was introduced instead of normal seawater after larval loading.

Image Acquisition, Extended. Four-dimensional imaging data (time, fluorescence channels, and z-slices) of Aiptasia larvae in the microfluidic device traps were acquired on a Leica DMI6000B stand equipped with a Yokogawa CSU-10 spinning disk head, QuantEM camera (Photometrics), and ASI MS2000 motorized XY stage under 20X magnification (Leica 20X/0.7 NA multi-immersion, used with glycerine solution). Z-stacks were collected in transmitted light and chlorophyll autofluorescence channels (561 nm excitation, 405/488/561 nm dichroic and 637/37 nm emission, Semrock) under control of SlideBook 6 (Intelligent Imaging Innovations). Where noted, detailed time-series focusing on one trap were collected in the transmitted light channel only.

Image Analysis, Extended. Images were analyzed in Fiji (ImageJ)(2). For algal fluorescence quantification, images in the time series were z-projected from the z-stack by sum intensity on the chlorophyll auto-fluorescence channel and minimal intensity on transmitted light; data in **Fig. 6b** is generated from mean fluorescence values (arb. units) extracted from equal size ROIs in the animal, expulsion area and background per frame from sum projection image in the algal fluorescence channel for each frame. For simple visualization, mid-plane z-slices in both channels were displayed and false-colored by transmitted light (grayscale) and chlorophyll autofluorescence (Lookup Table: Green Fire Blue) to create time-course images and movies.

Open Data Access. An open-source project repository for design files, imaging data for all trials (compressed as .avi image sequences), protocols, and additional resources, as mentioned in the text, is available at our **Open Science Framework** page, which can be found at OSF under [doi: 10.17605/osf.io/j2rsy].

Extended Notes

Bead Loading (Extended). During the bead capture trials displayed in **Fig. 3** and **Fig. S3**, larger beads appeared to clog at the inlet, likely due to steric restrictions imposed by the 23G hole punch. These occasional clogs, combined with size poly-dispersity of the beads and lack of bead deformation, complicated efforts to accurately model trapping of large, deformable organisms. Therefore, these measurements can be used as approximate loading conditions for a variety of different organism sizes beyond those characterized in smaller cell literature, but should be supplemented with empirical testing with the chosen organism.

Seawater Retention (Extended). The initial 4 frames within **Fig. 4** (time: 0-120 min) images were acquired before a change in room lighting; remaining images were acquired immediately following that first capture with no additional changes. Images displayed in this figure therefore contain a difference in background intensity due to this change in local lighting conditions.

Device Operation Protocol & Troubleshooting

Device Operation Protocol. We used the following protocol for conducting Aiptasia imaging experiments. This protocol could easily be translated to other organisms, if desired.

1. Set up the device as shown in [**Fig. S1**]. Load a syringe with desired nutrient solution (*e.g.* seawater) and turn on the syringe pump to 100 µL/min. Plug in the device inlet connections and open the inlet and outlet stopcocks to allow for flow through the trapping array.

2. Close the outlet stopcock and wait for ~2 minutes for the device to de-bubble. During this time, visually inspect the device and confirm all bubbles have left the trapping area before proceeding. Depending on the thickness of your PDMS device, de-bubbling can require up to 5 minutes. After all bubbles have left the traps, wait 10 s and open the outlet stopcock.

3. Flow the nutrient solution for 1 min at 100 µL/min and prepare for cell loading.

4. When ready, close the inlet stopcock, stop the syringe pump, and load 50 µL cells or organisms at the desired density (~1000 Aiptasia larvae) into the Tygon tubing connected to the syringe. Reconnect the connections, being mindful to avoid bubbles.

5. Wait up to 2 minutes for organisms to concentrate in the loading tubing upstream of the stopcock. Afterward, resume flow and open the inlet stopcock.

6. Begin imaging.

Other sample-loading loops and/or multiple syringe pumps in series can be used for exchanges as described in Step 4, if desired. This protocol and accompanying setup represents one cost-effective solution but is by no means meant to be restrictive or authoritative. Most importantly, stopcocks or similar mechanisms must be used during de-bubbling and loading to mitigate bubbles and organism loss during device preparation and buffer exchange in any protocol.

Bubble Reduction and Troubleshooting. In troubleshooting larval loading into the microfluidic device, we found bubble nucleation due to sample line exchanges and low flow rates to be dominant sources of reduced Aiptasia capture, trapping retention, or, in the case of massive bubble nucleation, premature larval death. We recommend the following guidelines for operation:

- Flow rates for the duration of the experiment should be set as high as possible (typically >20 µL/min). High flow rates keep the chamber fully expanded at all times, preventing bubbles from traveling through PDMS. These high flow rates also aid in reducing back-swimming in motile organisms, from our observations with Aiptasia. Early trials (such as those in **Fig. 4, 6, S5, S6, S9**) were conducted at 35 µL/min, which had low bubble nucleation, but occasional bubble nucleation was still observed even after de-bubbling. Later trials (such as those in **Fig. 5**) used 100 µL/min flow rates and the full stopcock assembly as shown, and no bubble nucleation was observed after initial debubbling. We recommend this higher flow rate for ease-of-assay.

- An in-line bubble trap with a stopcock for switching reagents or input solutions should be used to eliminate in-line bubbles during sample or syringe exchanges. We recommend the system used here, but more sophisticated sample-loading loops or systems are compatible as well.

- Syringe pumps should be aligned vertically above the sample plane when loading large organisms to prevent organism settling in horizontal positions in the loading tubing.

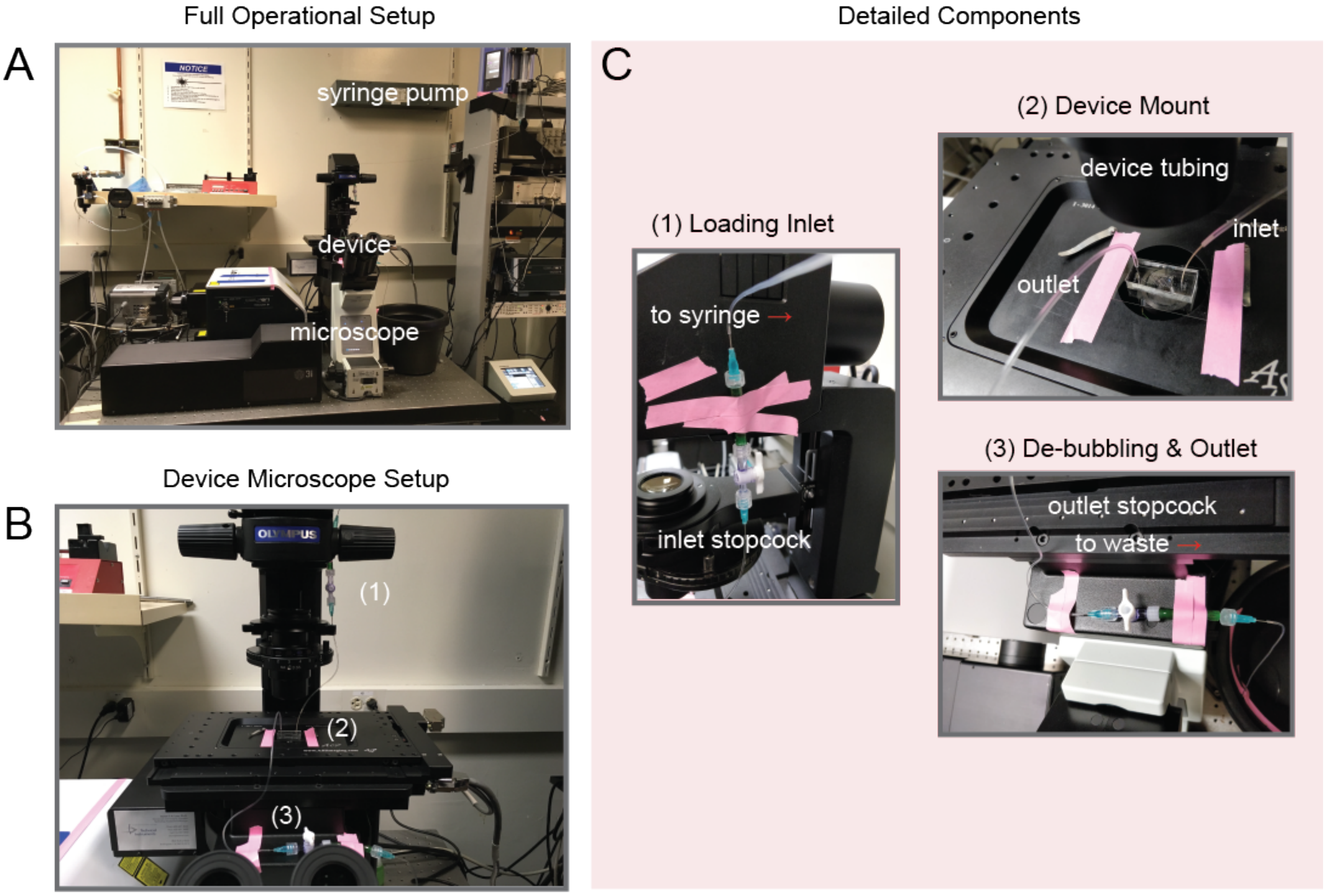

Fig. S1. Experimental Setup Overview for Aiptasia Trapping Experiments. The microfluidic device is positioned on the imaging platform and animals and reagents are introduced via a syringe pump above the device plane connected to an inlet stopcock assembly in-line with device tubing to the inlet, as shown. A similar device outlet stopcock assembly from the device outlet to waste is used for de-bubbling the device. An operating protocol is described in *Supplemental Information* and a parts list is available in **[Table S2]**.

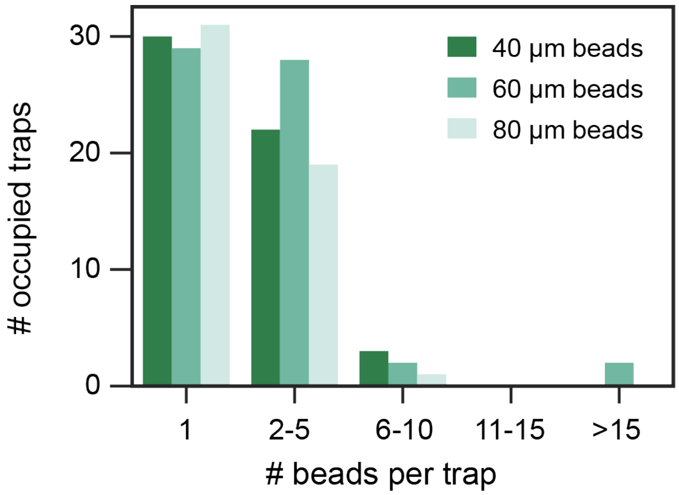

Fig. S2. Distribution of bead counts within occupied traps from bead-loading tests for 40 (dark green), 60 (green), and 80 (light green) µm beads for the empirically chosen 90 µm chamber height and 20 µm trap aperture geometry. Note the absence of major bead clusters at all bead sizes.

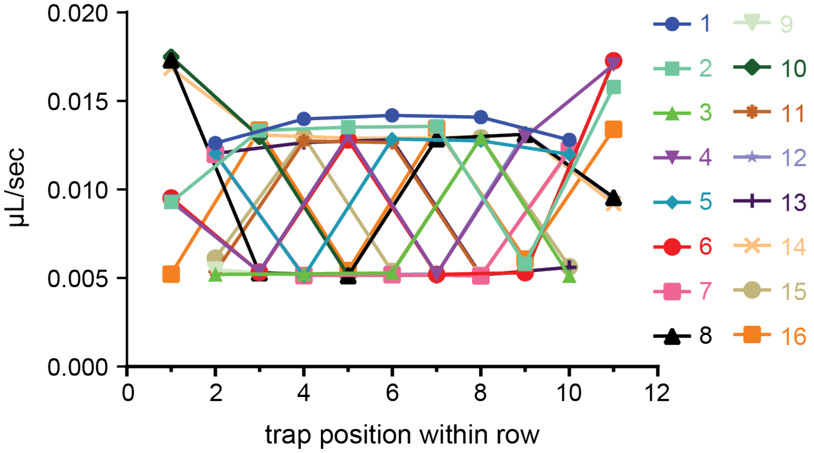

Fig. S3. Simulated flow rates within a random distribution of occupied and unoccupied traps. Flow rates of 0.012-0.013 μL/s (top cluster) and 0.005-0.006 μL/s (bottom cluster) are achieved in unoccupied and occupied traps, respectively. These data establish differences in flow as a function of position across the trapping array are minimal.

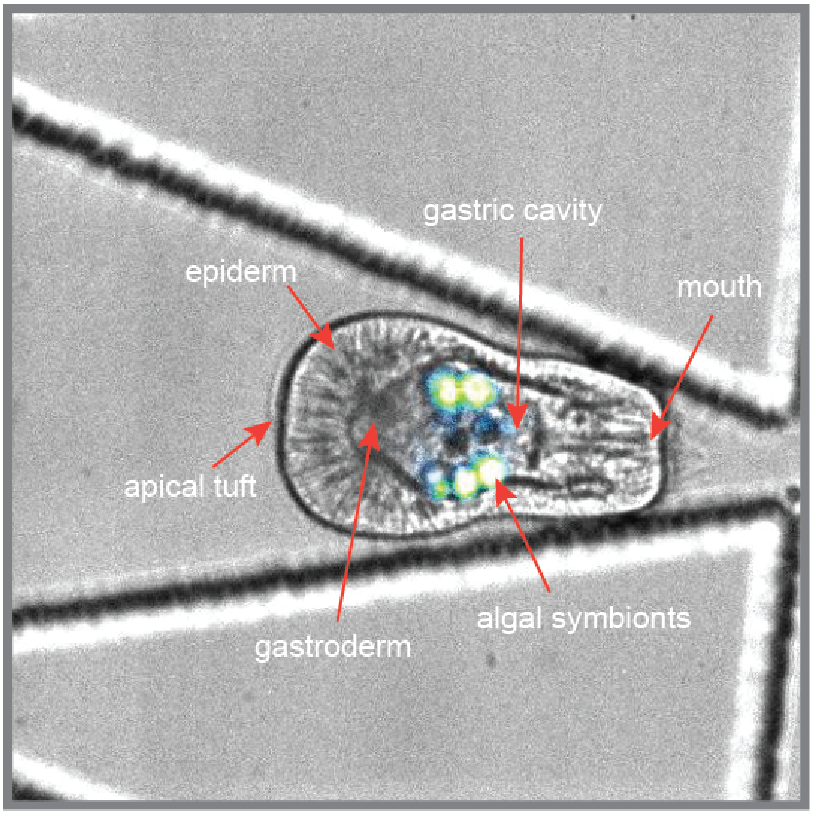

Fig. S4. Detailed view of a live, trapped Aiptasia larva and algal symbionts as captured by confocal microscopy (mid-plane transmitted light with merged far-red algal autofluorescence channel false colored to blue-green) under seawater flow with organism anatomy denoted by arrows. Note that the gastroderm layers over the gastric cavity in three-dimensional space. In this image, algal symbionts reside in the gastroderm of the organism.

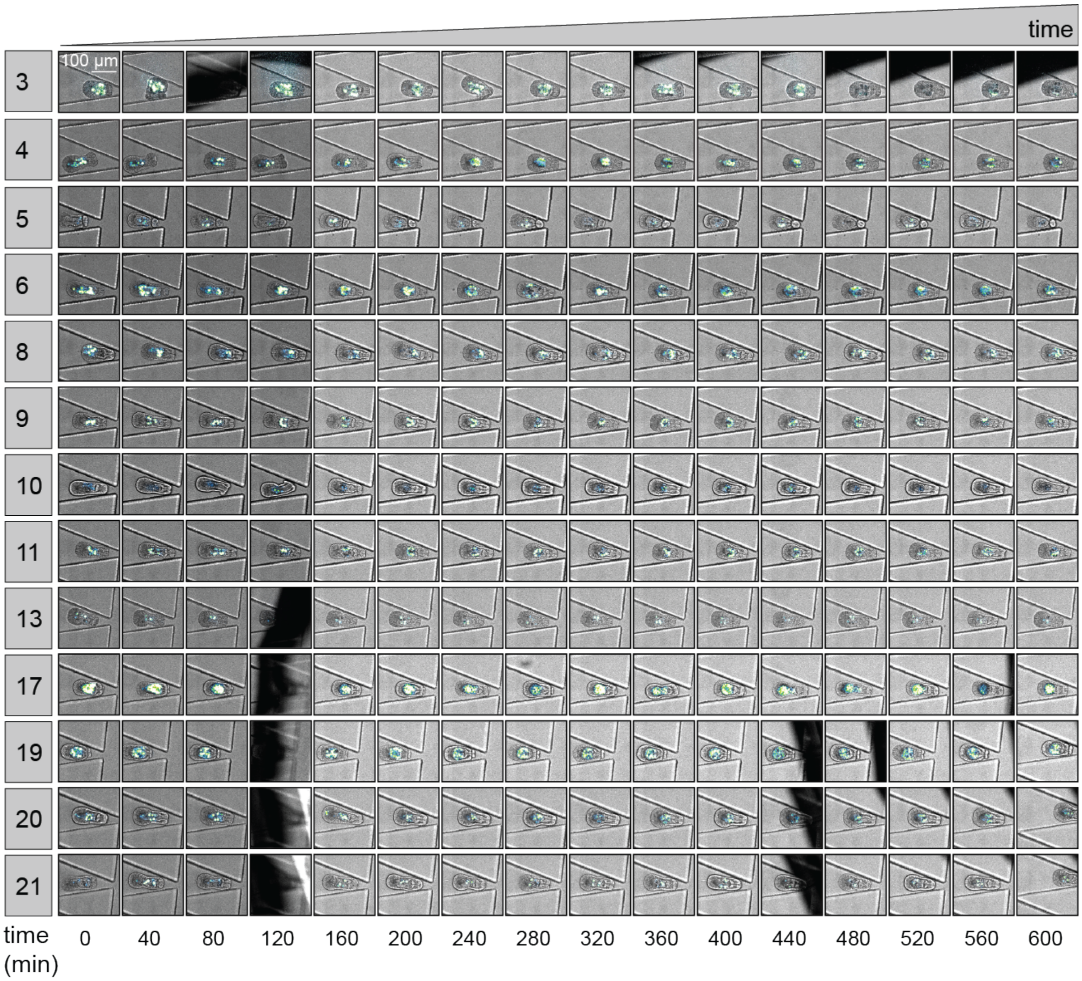

Fig. S5. Imaging time course for first 13 traps of 25 occupied traps in seawater trapping experiments referenced in **Fig. 4** (TrapIDs: 3-21) containing single trapped Aiptasia demonstrating stable trapping over 10 hours. Anomalous dark regions are due to tubing from the microfluidic device pulled into the imaging area during this experiment.

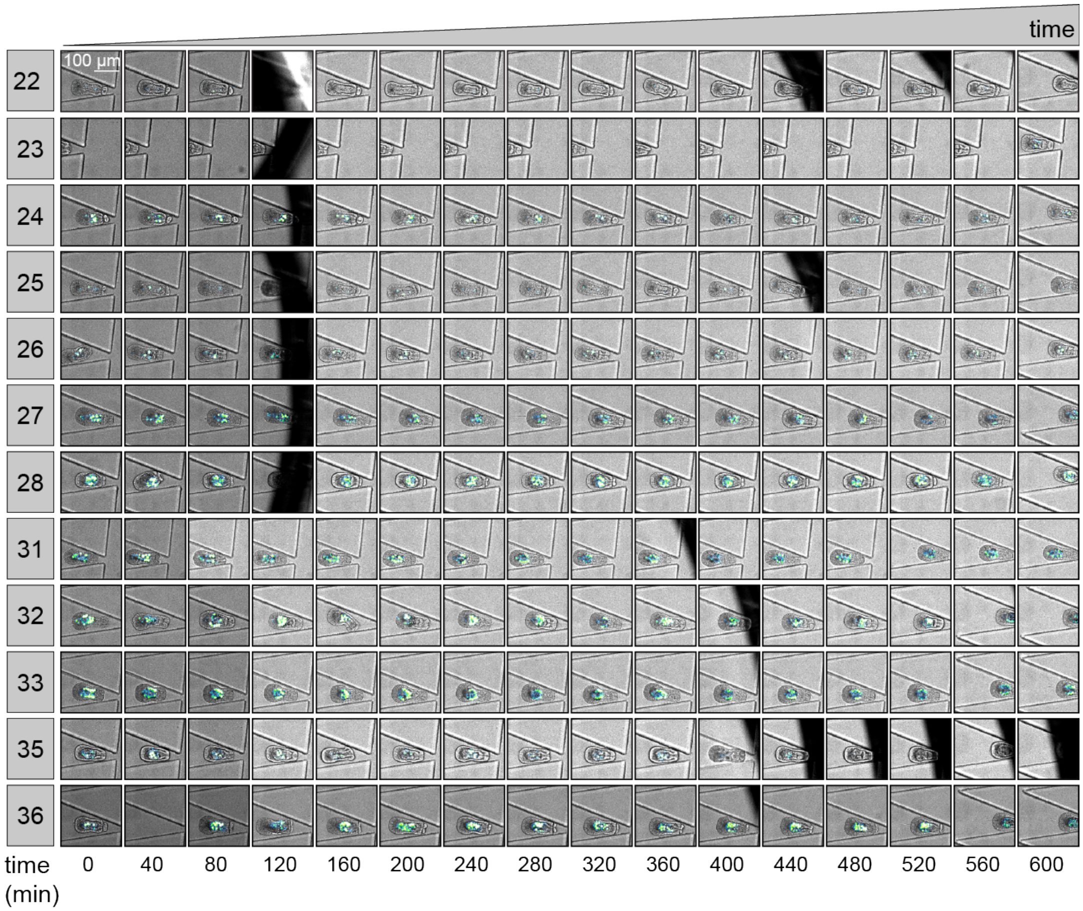

Fig. S6. Imaging time course for second 12 traps of 25 occupied traps in seawater trapping experiments referenced in **Fig. 4** (TrapIDs: 22-35) containing single trapped Aiptasia demonstrating stable trapping over 10 hours. Anomalous dark regions are due to tubing from the microfluidic device pulled into the imaging area during this experiment.

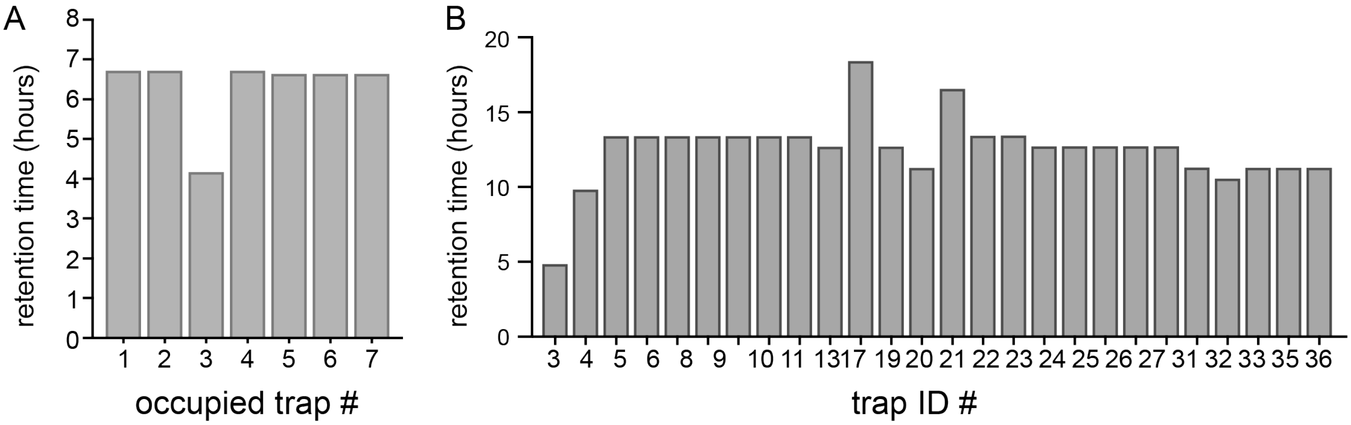

Fig. S7. Aiptasia retention times within traps. (*A*) Low-density loading experiment(∼100 larvae loaded) with 7 occupied traps. Flow at 100 µL/min proceeded for ∼6 hours after loading. (*B*) High-density loading experiment (∼1000 larvae loaded) with 24 occupied traps. Flow at 35 µL/min proceeded for ∼11.5 hours after loading.

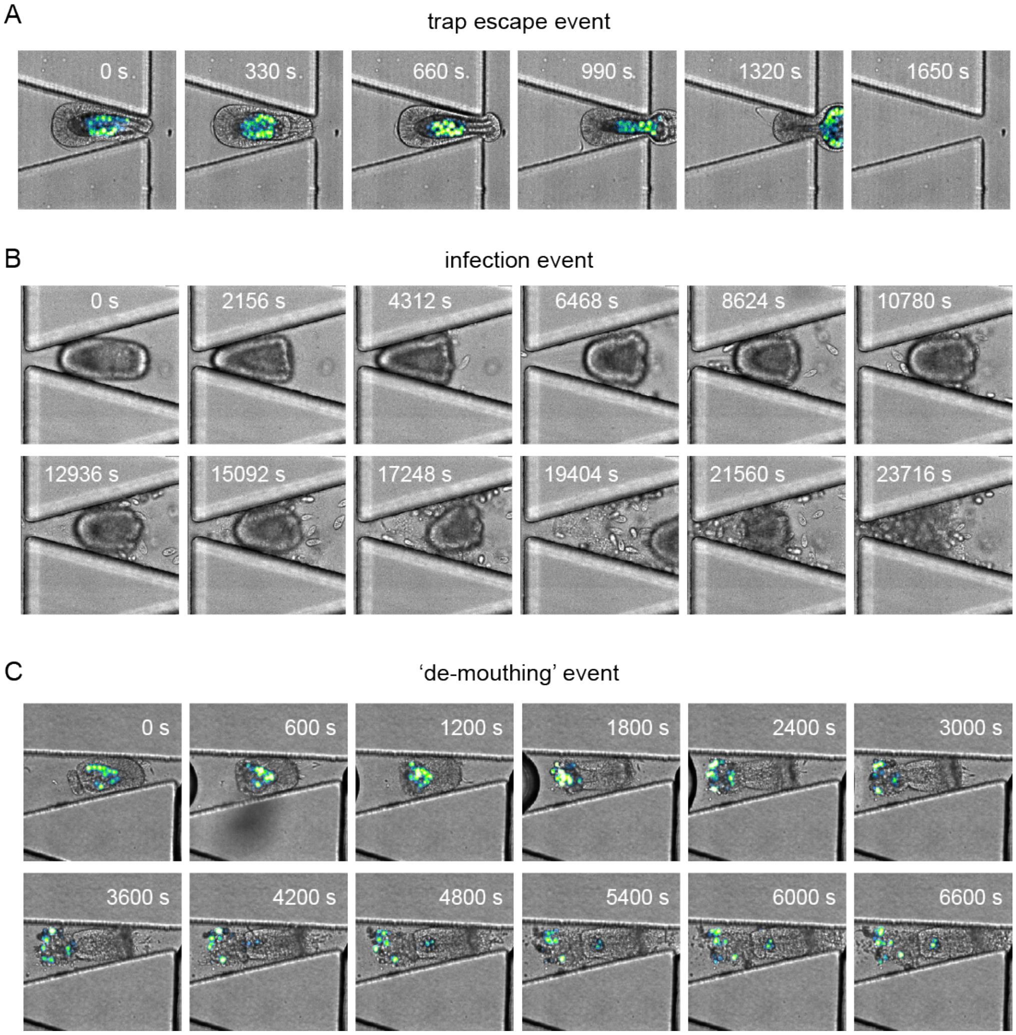

Fig. S8. Unusual observed trapped Aiptasia phenotypes. (*A*) Trap escape event showing extensive deformation followed by extrusion through aperture. (*B*) Aiptasia death due to ciliate infestation, likely acquired in dish culture or during algal infection. (*C*) Aiptasia death event due to a local bubble nucleation at low flow rate. The larva appeared to separate the mouth organ from the gastric cavity prior to disintegration.

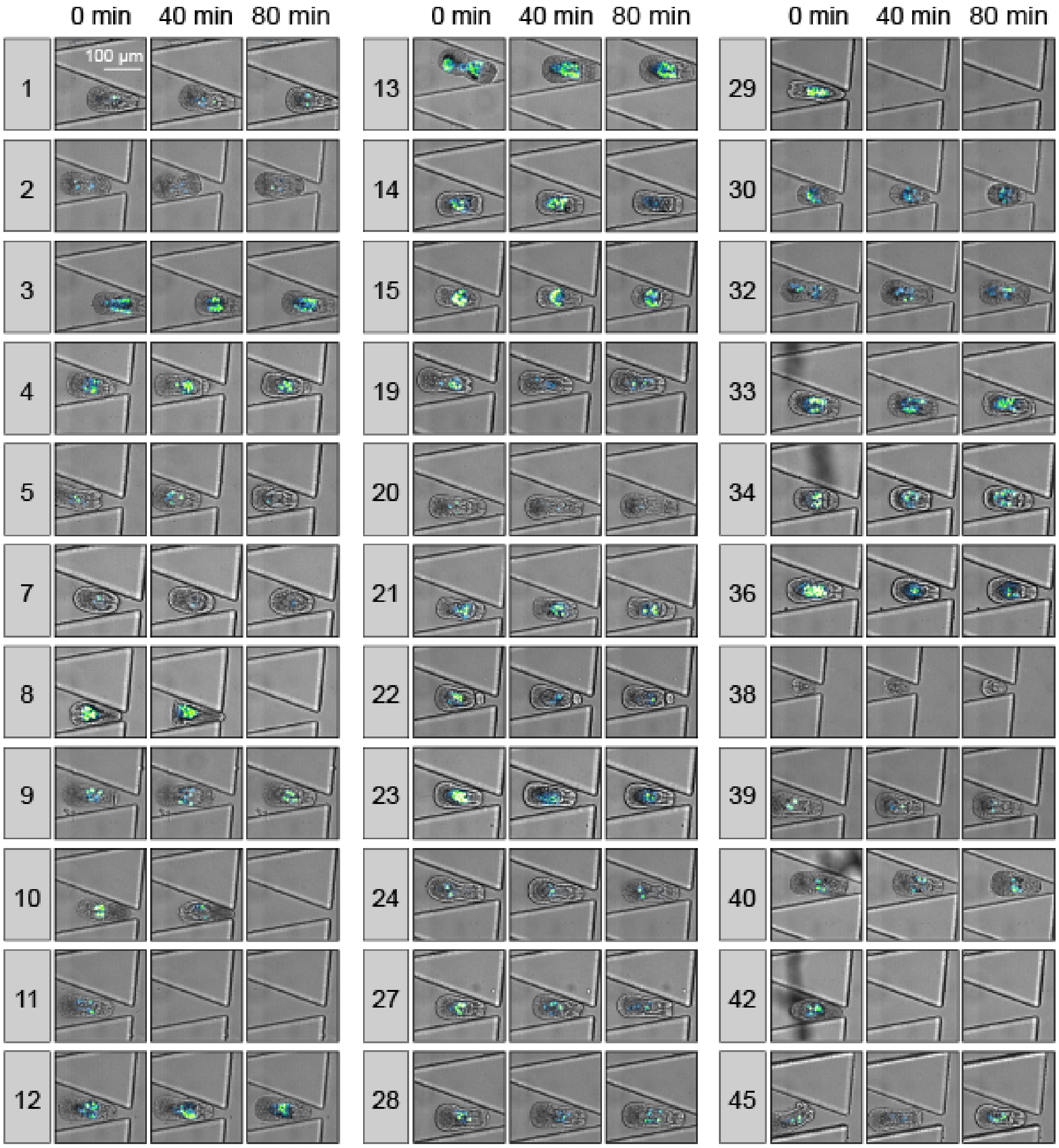

Fig. S9. Imaging time course showing trapped Aiptasia exposed to DCMU over 80 minutes. Aiptasia appeared to escape traps more often in the presence of DCMU.

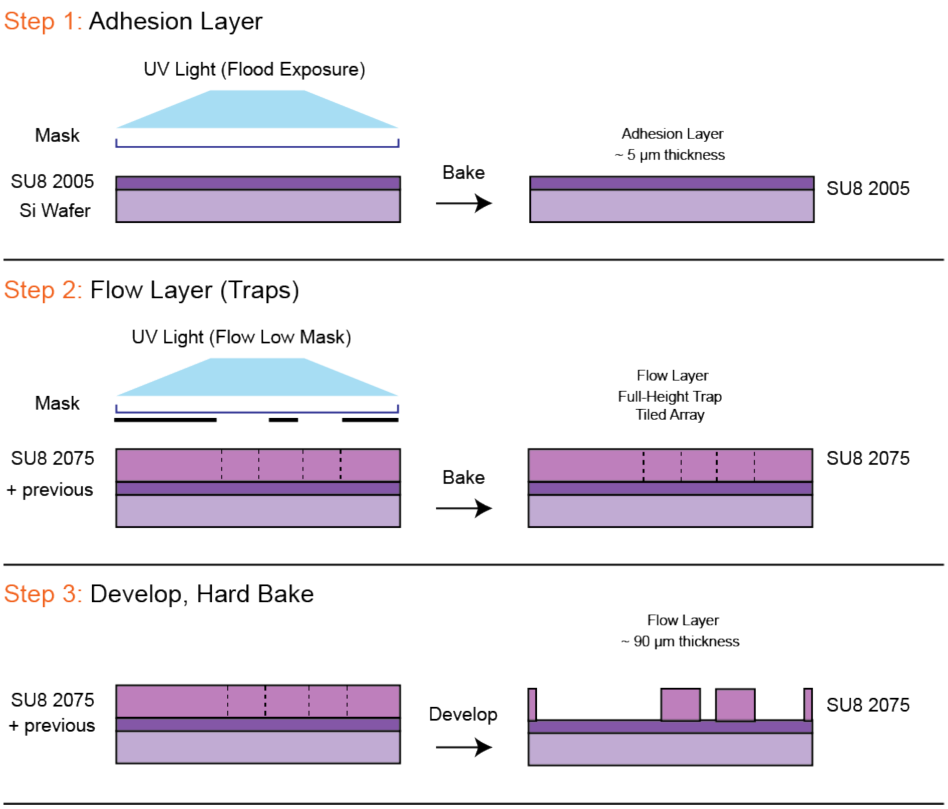

Fig. S10. Fabrication diagram illustrating the photolithography process. Further details are described in Extended Methods and outlined in **[Table S3]**.

Table S1. COMSOL Parameters, Extended. All parameters for replicating nutrient flow simulations presented in [Fig. 2].

**Operation Type**	**Parameter**	**Setting**
Global Settings	Module	Laminar Flow
	Material	Water
	Entrance Length	6 mm
	Outlet Pressure	1 atm.
Inlet Flow	Inlet Width	0.8 mm
	Inlet Height	0.09 mm
	Inlet Area	0.072 mm^2
	Vol. Flow Rate	100 µL/min
Streamline Generation	Vol. Flow Rate	100 µL/min
Aiptasia Rigid Body Inlet Flow	Inlet Width	0.8mm
	Chamber Height Modeled	[50.0, 70.0, 90.0] µm
	Semi-axis (a)	[63.0, 52.5, 46.6] µm
	Semi-axis (b)	[98.0, 81.8, 72.5] µm
	Semi-axis (c)	[23.0, 33.0, 42.0] µm
	Body Volume (mm^3)	0.000595 $µm^3$, all heights
	Body Volume (µL)	0.000595 µL, all heights

Table S2. Microfluidic Device Assembly Recommended Parts. These items, denoted in bulk, construct the inlet and outlet stopcock assemblies as well as the tubing connections for operating the 'Traptasia' microfluidic device. Bulk costs reflect the cost of $\sim$ 500 experiments. Stopcock assemblies should be constructed according to [Fig. S1] and are reusable for each experiment. Tygon and PEEK tubing should be replaced each experiment.

Component	Source	Catalog Number	Quantity	Cost Per Unit	Link
1/4" OD x 1/8" ID polyurethanetubing, green, 50 ft rool	McMaster-Carr	5648K74	1	$29.00	Link
Tygon ND-100-80 Tubing (500' roll, 0.06" OD, 0.02" ID)	Fisher Scientific	14-171-284	1	$317.00	Link
Male Luer Integral Lock Ring, 1/8" barb, Pack of 100	Value Plastics	MTLL230-9	1	$20.20	Link
Steel Blunt Pins, 1/2" length, Pack of 250	New England Small Tube	NE-1310-02	1	$212.50	Link
1-Way Luer Stopcock, M Lock, Pack of 100	Cole-Parmer	Sk-30600-05	1	$185.00	Link
Blunt Stainless Steel Luer Lock Needle, Pack of 50	McMaster-Carr	75165A684	1	$11.88	Link
PEEK Tubing, 25 ft.	Zeus	Custom	1	$75.00	0.02" OD, 0.01" ID
Off-Chip Bubble Trap	Darwin Microfluidics	KBTLarge	1	$147.37	Link

Table S3. Fabrication Protocol and Parameters, Extended. Steps (A) and (B) remain the same for all chamber heights; Step (C) is chosen for the chamber height desired. [50, 70, 90] µm chamber heights were explored in this work, with 90 µm devices used for all larval imaging experiments

Step Num.	Description	Parameters
**A**	**Dehydration bake**	**95℃ for 10 mins**
**B**	**SU-8 2005 Adhesion Layer**	
B.1	Spin SU-8 2005	
	Spread	500 rpm, 10 s, 133 rpm/s acc
	Cast	3000 rpm, 40 s, 266 rpm/s acc
B.2	Soft bake	65℃ for 2 min, 95℃ for 3 min, 65℃ for 2 min
B.3	Expose without mask	20 s at 5.7 mW/cm^2
B.4	Post-exposure bake	65℃ for 2 min, 95℃ for 4 min, 65℃ for 2 min
**C**	**SU-8 2075 for Flow Square**	**Mask: Single Layer 90 µm height**
C.1	Spin SU-8 2075	
	Spread	500 rpm, 10 s, 133 rpm/s acc
	Cast	2100 rpm, 40 s, 266 rpm/s acc
	Edge bead removal	3400 rpm, 1 s, 3000 rpm/s acc
C.2	Photoresist relaxation	10 minutes
C.3	Soft bake	65℃ for 2 min, 95℃ for 22 min, 65℃ for 2 min
C.4	Expose	41.2 s @ 5.6 mW/cm^2 (230 mJ)
C.5	Post-exposure bake	65℃ for 5 min, 95℃ for 12 min, 65℃ for 5 min
C.6	Develop	SU-8 developer
C.7	Hard bake	65℃-165℃ at 120℃/hr, 2.5 hr timer, auto-off
**C**	**SU-8 2075 for Flow Square**	**Mask: Single Layer 70 µm height**
C.1	Spin SU-8 2075	
	Spread	500 rpm, 10 s, 133 rpm/s acc
	Cast	3250 rpm, 40 s, 266 rpm/s acc
	Edge bead removal	3400 rpm, 1 s, 3000 rpm/s acc
C.2	Photoresist relaxation	20 minutes
C.3	Soft bake	65℃ for 2 min, 95℃ for 15 min, 65℃ for 2 min
C.4	Expose	37.4 s @ 5.5 mW/cm^2 (205.71 mJ)
C.5	Post-exposure bake	65℃ for 5 min, 95℃ for 8 min, 65℃ for 5 min
C.6	Develop	SU-8 developer
C.7	Hard bake	65℃-165℃ at 120℃/hr, 2.5 hr timer, auto-off
**C**	**SU-8 2050 for Flow Square**	**Mask: Single Layer 50 µm height**
C.1	Spin SU-8 2050	
	Spread	500 rpm, 10 s, 133 rpm/s acc
	Cast	3200 rpm, 40 s, 266 rpm/s acc
	Edge bead removal	3400 rpm, 1 s, 3000 rpm/s acc
C.2	Photoresist relaxation	10 minutes
C.3	Soft bake	65℃ for 2 min, 95℃ for 8 min, 65℃ for 2 min
C.4	Expose	29.86s @ 5.3 mW/cm^2 (159.28 mJ)
C.5	Post-exposure bake	65℃ for 5 min, 95℃ for 8 min, 65℃ for 5 min
C.6	Develop	SU-8 developer
C.7	Hard bake	65℃-165℃ at 120℃/hr, 2.5 hr timer, auto-off

Movie S1. Aiptasia larvae visualized in dish culture under a stereoscope (10 ms exposure, 14.76 s clip, shown at 10 fps).

Movie S2. Example Z-Stack Imaging of a Trapped Aiptasia Larva and Algal Symbionts in Seawater. The z-stack shows the full extent of the larval body in the trap. While data is displayed as mid-plane in this report, z-stacks are available for each trapped larva and may be relevant for different stress-related phenotypes.

Movie S3. Example Time Course (Capture 1) of a Trapped Aiptasia Larva and Algal Symbionts in Seawater, transmitted light only. Time-lapse series was acquired at 50 ms transmitted light exposure with 5 z-slices, shown at a single mid-plane, over 20 minutes (shown at 10 fps). Note the ability to resolve larval spin and individual algal movement. This movie is an expansion of [Fig. 5]. Flow rate is relaxed from 125 µL/min to 100 µL/min in first frames of the sequence.

Movie S4. Example Time Course (Capture 3) of a Trapped Aiptasia Larva and Algal Symbionts in Seawater, transmitted light only. Time-lapse series was acquired at 50 ms transmitted light exposure with 5 z-slices, shown at a single mid-plane, over 20 minutes (shown at 10 fps). Note the ability to resolve larval spin and individual algal movement. Differences between different larva can be seen when comparing this time-lapse to [SI Movie 2].

Movie S5. Observation of Loss of Aiptasia Larval Viability and Subsequent 'De-mouthing' Behavior in Seawater, transmitted light only. Images were acquired in the same fashion as [SI Movies 2, 3], except the time-lapse was acquired for >12 hours for the individual larva (shown at 100 fps).

Movie S6. Aiptasia Bleaching Event under 25 µM DCMU in Seawater. This movie is an expansion of [Fig. 6]. Note clear algal expulsion through the larval mouth at 95 min.

References

1. Brower K, White AK, Fordyce PM (2017) Multi-step Variable Height Photolithography for Valved Multilayer Microfluidic Devices. *Journal of Visualized Experiments* (119):1–12.
2. Schindelin J, et al. (2012) Fiji: an open-source platform for biological-image analysis. *Nature Methods* 9(7):676–682.
